# Exploration of three *Dyadobacter fermentans* enzymes uncovers molecular activity determinants in CE15

**DOI:** 10.1007/s00253-024-13175-6

**Published:** 2024-05-15

**Authors:** Miriam Carbonaro, Scott Mazurkewich, Gabriella Fiorentino, Leila Lo Leggio, Johan Larsbrink

**Affiliations:** 1 Department of Biology, University of Naples Federico II, 80126 Naples, Italy; 2 Wallenberg Wood Science Center, Division of Industrial Biotechnology, Department of Life Sciences, Chalmers University of Technology, SE-412 96 Gothenburg, Sweden; 3 Department of Chemistry, University of Copenhagen, Universitetsparken 5, DK-2100 Copenhagen, Denmark

**Keywords:** Lignocellulose, Biomass, Glucuronoyl esterase, Hydrolase, *Dyadobacter*

## Abstract

**Abstract:**

Glucuronoyl esterases (GEs) are serine-type hydrolase enzymes belonging to carbohydrate esterase family 15 (CE15), and they play a central role in the reduction of recalcitrance in plant cell walls by cleaving ester linkages between glucuronoxylan and lignin in lignocellulose. Recent studies have suggested that bacterial CE15 enzymes are more heterogeneous in terms of sequence, structure, and substrate preferences than their fungal counterparts. However, the sequence space of bacterial GEs has still not been fully explored, and further studies on diverse enzymes could provide novel insights into new catalysts of biotechnological interest. To expand our knowledge on this family of enzymes, we investigated three unique CE15 members encoded by *Dyadobacter fermentans* NS114^T^, a Gram-negative bacterium found endophytically in maize/corn (*Zea mays*). The enzymes are dissimilar, sharing ≤ 39% sequence identity to each other‚ and were considerably different in their activities towards synthetic substrates. Combined analysis of their primary sequences and structural predictions aided in establishing hypotheses regarding specificity determinants within CE15, and these were tested using enzyme variants attempting to shift the activity profiles. Together, the results expand our existing knowledge of CE15, shed light into the molecular determinants defining specificity, and support the recent thesis that diverse GEs encoded by a single microorganism may have evolved to fulfil different physiological functions.

**Key points:**

• *D. fermentans encodes three CE15 enzymes with diverse sequences and specificities*

• *The Region 2 inserts in bacterial GEs may directly influence enzyme activity*

• *Rational amino acid substitutions improved the poor activity of the DfCE15A enzyme*

**Supplementary information:**

The online version contains supplementary material available at 10.1007/s00253-024-13175-6.

## Introduction

Lignocellulosic residues from plant biomasses are an attractive feedstock to produce value-added materials due to their abundance, cost-effectiveness, and crucially, ready availability in the form of forestry and agricultural side streams. However, breaking down the plant cell wall components is a significant hurdle due to the robust network of cellulose fibers, hemicelluloses, and lignin, which renders lignocellulose resistant to deconstruction. A contributor to this recalcitrance is the prevalence of lignin-carbohydrate complexes (LCCs), covalent bonds formed during lignification between lignin and exposed polysaccharides, primarily hemicellulose (Agger et al. 2023). Four LCC linkages have been shown thus far: benzyl ether, benzyl ester (α- or γ-carbon), phenyl glycoside, and acetal linkages (Zhao et al. 2020). The hydrolysis of these stable linkages represents a significant hurdle and costly step in the conversion of lignocellulosic feedstocks into oligo- and monosaccharides, as well as in the overall delignification process. Glucuronoyl esterases (GEs, EC number 3.1.1.117) catalyze the hydrolysis of the ester linkage between lignin and glucuronoyl moieties in glucuronoxylan LCCs, thus playing an important role in the deconstruction of lignocellulose by metabolizing bacteria and fungi (Larsbrink and Lo Leggio 2023). GEs are thus far only found in carbohydrate esterase family 15 (CE15) in the Carbohydrate Active Enzymes database (http://www.cazy.org) (Drula et al. 2022), a family which contains considerable sequence diversity. While work from several groups in recent years has greatly advanced our understanding of the family (Agger et al. 2023; Larsbrink and Lo Leggio 2023; Monrad et al. 2018), several questions remain open concerning the diversity and structure-function properties of these important and industrially relevant enzymes.

GEs are serine hydrolases belonging to the α/β-hydrolase (ABH) superfamily. They comprise a catalytic triad (Ser-His-Glu/Asp) and, uniquely amongst ABH enzymes, a conserved arginine side chain shown to stabilize the oxyanion intermediate (Larsbrink and Lo Leggio 2023). The acidic residue of the triad has been observed in two positions: in the canonical ABH position at the end of strand β7 (or β8, depending on reference fold), found prominently amongst bacterial members, or in the non-canonical position at the end of strand β6 (or β7, depending on reference fold), found prominently amongst fungal members (Arnling Bååth et al. 2018; Ernst et al. 2020; Nardini and Dijkstra 1999). Some bacterial members, such as *Ot*CE15A from *Opitutus terrae*, contain acidic residues in both positions, and both of these have been shown to be functional (Arnling Bååth et al. 2018; Mazurkewich et al. 2019; Zong et al. 2022). The substrate binding cleft in GEs is located above the main β-sheet of the ABH fold which is found to be quite solvent exposed in fungal CE15 members, whereas additional inserted regions present in bacterial members result in a build-up around the cleft leading to a deeper pocket (Arnling Bååth et al. 2018, 2019; Ernst et al. 2020; Krska et al. 2021). Several structures of GEs with bound carbohydrate ligands have provided insights on their interactions with monosaccharide uronic acids, dominated by extensive hydrogen bonding; recently, it has also been shown that GEs can directly interact with larger glucuronoxylooligosaccharides, establishing van der Waals interactions with a conserved tryptophan residue in the binding cleft (Ernst et al. 2020; Mazurkewich et al. 2019; Seveso et al. 2024; Zong et al. 2022). Lignin fragments are proposed to be positioned opposite to the sugar binding site, in a region that is open and solvent exposed in fungal members but more encased in bacterial enzymes due to the presence of an additional insert referred to as Region 2, which is typically rich in aromatic residues and could be responsible for the interaction with the lignin side of the natural substrate (Larsbrink and Lo Leggio 2023).

While the specific targeting of certain GEs towards glucuronoxylan LCCs is well-established (Arnling Bååth et al. 2016; Ernst et al. 2020; Mosbech et al. 2019, 2018), there are indications suggesting that some members of the family may serve alternative purposes. For example, bacteria frequently possess more than one CE15 gene, with some studied species harboring up to three or four copies dispersed in their genome, which display differential gene expression in response to nutrient sources (Arnling Bååth et al. 2018; Drula et al. 2022). Some CE15 members from *Bacteroidota* species are situated in putative glucuronoxylan-targeting polysaccharide utilization loci (PULs)—gene clusters that encompass all the necessary genes for the regulation, transport, and degradation of specific polysaccharides (McKee et al. 2021)—characterized by the presence of a repertoire of xylanolytic enzymes encoded in close proximity where putative GEs would contribute by cleaving glucuronoxylan LCCs (Terrapon et al. 2018). In non-*Bacteroidetes*, CE15 members can be found in similar arrangements, i.e., in putatively co-regulated gene clusters. In a recent study, three CE15 enzymes from PULs proposed to target pectins rather than xylan were found to act on both galacturonate and glucuronate esters, implying a role for the enzymes in pectin or rhamnogalacturonan metabolism (Seveso et al. 2024; Shah et al. 2023). Additionally, some studied CE15 enzymes, such as *Lf*CE15C from *Lentithecium fluviatile* (Mazurkewich et al. 2023), *Ot*CE15B from *O. terrae* (Arnling Bååth et al. 2018), and *Be*CE15 from *Bacteroides eggerthii* (Kmezik et al. 2021), exhibited a complete lack of, or significantly compromised, activity on both model substrates and/or glucuronoxylan LCCs, despite the proteins’ predicted or experimentally determined structures indicating a compatibility with GE activity. This lack of, or diminished, activity suggests a specificity for substrates that are yet to be elucidated. Taken together, previous analyses suggest that additional biological roles for CE15 members remain undiscovered although they could be significant for bacterial catabolism of carbohydrate-rich biomass. Efforts to uncover the enzymes’ potential biological roles could enhance our understanding of these enzymes which could in turn be instrumental for engineering endeavors aimed at improving their industrial applications in lignocellulose valorization.

The objective of this study was to examine the CE15 members from the Gram-negative bacterium *Dyadobacter fermentans* NS114^T^, a type species of the *Dyadobacter* genus within the *Bacteroidota* phylum. This endophytic species was originally isolated from healthy *Zea mays*, suggesting a neutral symbiosis with its plant host (Chelius and Triplett 2000). *D. fermentans* encodes three CE15 members (*Df*CE15A, *Df*CE15B, and *Df*CE15C), each located in an unexplored region of the phylogenetic tree (described in more detail below), which may have roles different from the typical LCC-cleaving GEs (Lang et al. 2009). The genetic neighborhood of each gene was assessed, and a function in LCC valorization is suggested for only one of the enzymes. The *Df*CE15 proteins were heterologously produced and characterized on synthetic uronic acid ester substrates revealing a distinct activity profile for each. The predicted structures of the enzymes were analyzed, and the role of different amino acid residues in their substrate-binding pockets was assessed by constructing enzyme variants and comparing their activity with previously characterized CE15 members. Collectively, this study sheds light on the diversity in CE15, particularly highlighting differences in members from previously unexplored regions of the family’s phylogenetic tree, and paves a path for future inquiries including activities towards recalcitrant lignocellulose and possible additional metabolic activities.

## Materials and methods

### Bioinformatics

The phylogenetic tree was created using all the CE15 entries present in CAZy (May 23rd, 2023) with fragment sequences and duplicated/identical sequences removed. The remaining sequences (*n* = 604) were trimmed to comprise only the catalytic domain, aligned using ClustalOmega (Madeira et al. 2022), and the tree was constructed using IQ-TREE (Minh et al. 2020) with automatic identification of substitution model (WAG + F + I + G4), 1000 ultrafast bootstraps, and visualized using iTOL (Letunic and Bork 2021). The genomic neighborhood of each *D. fermentans *CE15 gene was manually curated through protein sequence analysis of each gene ~ 10–20 genes upstream and downstream of the CE15 member. The closest characterized homolog for a neighboring gene was identified through either primary sequence analysis using BLAST (Altschul et al. 1990) or structural analysis through Foldseek (van Kempen et al. 2023) from the AlphaFold2 (Varadi et al. 2022) predicted structure found on Uniprot (Consortium 2023). Protein structural comparisons used AlphaFold2 models, accessible through Uniprot (Uniprot entry C6W6A8 for *Df*CE15A; C6W6K0 for *Df*CE15B; and C6VY01 for *Df*CE15C), and structural alignments were created in PyMOL 2.5 (Schrödinger-LLC 2023).

### Molecular biology and protein production

CE15 genes (loci tags DFER_RS15000, DFER_RS15475, DFER_RS28515 for *Df*CE15A, *Df*CE15B, and *Df*CE15C, respectively), lacking coding regions corresponding to their predicted signal peptides, were amplified by PCR from genomic DNA of *Dyadobacter fermentans* NS114^T^ using the primers shown in Supplemental Table S1. Each amplified product was cloned into a Novagen pET-28a vector (Merck Millipore, Burlington, MA, USA), containing a N-terminal His_6_ tag, and subsequently transformed into *Escherichia coli* HST08 Stellar cells (Clontech Laboratories, Mountain View, CA, USA; genotype: F-, *endA1*, *supE44*, *thi-1*, *recA1*, *relA1*, *gyrA96*, *phoA*, Φ80d *lacZ*Δ *M15*, Δ(*lacZYA*-*argF*) U169, Δ(*mrr-hsdRMS-mcrBC*), Δ*mcrA*, λ-). Constructs were confirmed by DNA sequencing (Eurofins Genomics, Galten, Denmark) and subsequently transformed into *E. coli* BL21 (λDE3) (Thermo Fisher Scientific, Waltham, MA, USA; genotype: F- *ompT hsdS*_*B*_ (r_B_–, m_B_–) *gal dcm*) for protein production.

Transformed cells were grown at 37 °C in lysogeny broth (LB medium) supplemented with 50 µg/mL neomycin at 150 rpm shaking for 3 h. Protein expression was then induced by addition of isopropyl β-d-1-thiogalactopyranoside (IPTG) to a final concentration of 0.2 mM and the cells incubated at 16 °C overnight. Cells were harvested by centrifugation at 18,000 × g for 15 min and subsequently resuspended in 50 mM tris(hydroxymethyl)aminomethane (TRIS) buffer pH 8 with 250 mM NaCl, 5 μg/mL lysozyme, and 10 µg/mL DNase I. Cells were lysed by sonication, and the soluble phase was collected by removing cell debris with centrifugation at 18,000 × g for 20 min. Enzymes were purified by immobilized metal ion affinity chromatography using 5-mL HisTrap Excel columns on an ÄKTA system (Cytiva, Uppsala, Sweden). The binding buffer comprised 50 mM TRIS pH 8 containing 250 mM NaCl, and the elution was performed using the binding buffer with the addition of 250 mM imidazole. Eluted enzymes were buffer exchanged by ultrafiltration using Amicon® Ultra-15 centrifugal filtration units (Merk Millipore, Burlington, MA, USA) and stored at 4 °C (*Df*CE15A and *Df*CE15C into 50 mM TRIS pH 8 buffer containing 100 mM NaCl and *Df*CE15B into 50 mM Na-citrate pH 6 buffer containing 100 mM NaCl). Sodium dodecyl sulfate polyacrylamide gel electrophoresis (SDS-PAGE) using Mini-PROTEAN TGX Stain-Free Gels (Bio-Rad, Solna, Sweden) was used to assess protein purity. Protein concentrations were determined using a Nanodrop 2000 Spectrophotometer (Thermo Fisher Scientific, Waltham, MA, USA) using the respective protein construct’s extinction coefficient. Enzyme variants *Df*CE15A-3v and *Df*CE15B-Q318W were created by site-specific mutagenesis using the QuikChange method using the primers listed in Supplemental Table S1 and were produced and purified in the same way as the wild-type proteins.

### Biochemical characterization

The uronoyl esterase activity of *Df*CE15A, *Df*CE15B, and *Df*CE15C and mutated variants was assayed by continuously monitoring the formation of uronic acid using the K-URONIC kit (Megazyme, Dublin, Ireland). The pH optimum for each enzyme was determined with 2 mM benzyl-d-glucuronate (BnzGlcA) as a substrate in a constant ionic–strength three-component buffer containing 50 mM TRIS–HCl, 25 mM acetic acid, and 25 mM 2-(*N*-morpholino) ethanesulfonic acid (MES), covering a pH range of 4.5–9.5 (Ellis and Morrison 1982). The kinetic measurements were performed using BnzGlcA, methyl-d-glucuronate (MeGlcA), and methyl-d-galacturonate (MeGalA), in 200-µL reactions containing 50 mM sodium phosphate pH 7.5, 2 µL uronate dehydrogenase, and 16 µL NAD^+^, as previously described (Arnling Bååth et al. 2018). The substrates were dissolved in 100% dimethyl sulfoxide (DMSO), and all reactions contained ≤ 10% DMSO, an amount determined to not affect the enzyme reactions.

### Determination of melting temperatures

The melting temperatures of the *Df*CE15 proteins were determined similarly to another recently characterized CE15 member (Krska and Larsbrink 2020). Briefly, thermal shift assays contained 5–10 μM of purified enzymes which were mixed in a 1:10 ratio with 5 × SYPRO Orange dye (Sigma-Aldrich, St. Louis, MO, USA) in a final volume of 30 μL. The reactions were transferred to a 96-well PCR plate, and melting curves were generated in a Stratagene Mx3005P Q-PCR machine (Agilent Technologies, Santa Clara, CA, USA) with a temperature gradient of 1 °C/min from 25 to 90 °C and quantifying the fluorescence at 516 nm. For each protein, the melting temperature (*T*_*m*_) was determined from the inflection point of the second derivative of the fluorescence response data.

## Results

### CE15 phylogeny

**Fig. 1:**
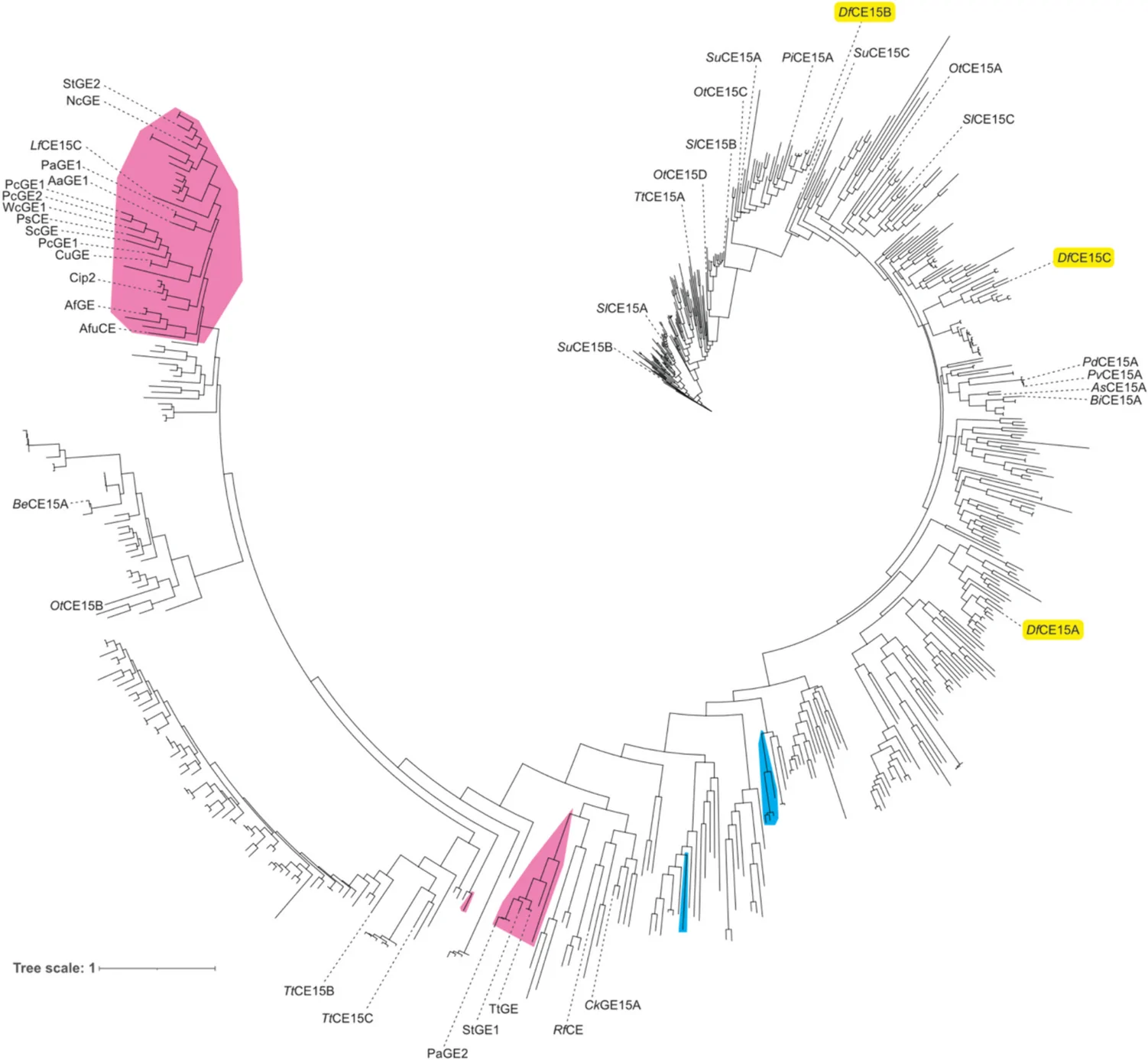
Phylogenetic tree of all CE15 catalytic domains in the CAZy database. Fungal entries are highlighted in fuchsia and archaeal in blue, and unshaded branches represent bacterial protein sequences. Proteins which have been studied in the literature are annotated, and the enzymes from *D. fermentans* are highlighted in yellow. Noteworthy is the breadth of diversity particularly among bacterial CE15 members which has yet to be investigated, with *Df*CE15A and *Df*CE15C representing previously unexplored clades

In a previous study in 2018 (Arnling Bååth et al. 2018), a phylogenetic tree of all CE15 members annotated in the CAZy database at the time (233 entries) was prepared to guide our investigation into the diversity within the family. Since then, the work of several groups has resulted in the investigation of many more CE15 members, effectively more than doubling the number of characterized enzymes within the family (see for instance recent reviews: Agger et al. 2023; Larsbrink and Lo Leggio 2023)). During this time, the identification of new CE15 members has also increased considerably (733 entries as of May 2023), especially for bacterial members. Therefore, a new phylogenetic tree was produced, resembling the older version with fungal and bacterial enzymes quite distinctly separated, but also revealing that although many CE15 members have now been characterized, many branches of the family tree remain unexplored (Fig. 1). For example, the three CE15 members from *D. fermentans* are found spread across the bacterial portion of the tree with both *Df*CE15CA and *Df*CE15C found in clades distant from characterized enzymes. *Df*CE15B, on the other hand, is found within a clade containing the characterized GEs *Pi*CE15A (Seveso et al. 2024), *Su*CE15A (Arnling Bååth et al. 2018), and *Ot*CE15C (Arnling Bååth et al. 2018). Thus, in our pursuit of a more comprehensive understanding of the family, the three CE15 enzymes encoded by *D. fermentans* represent interesting targets to further scrutinize activities and structure-function relationships within the family.

### Genomic neighborhoods surrounding the *D. fermentans* CE15 enzymes

Several bacterial species encode multiple putative CE15 members within their genome, a biological characteristic that remains not fully understood (Arnling Bååth et al. 2018). Bacteria commonly arrange genes essential for multi-step processes in proximity, facilitating co-transcription and/or regulation, as observed in some metabolic gene clusters like polysaccharide utilization loci (Foley et al. 2016; Martens et al. 2009; McKee et al. 2021). Thus, the neighborhood in which genes are situated can provide insights into the potential biological roles of enzymes. This phenomenon has not yet been explored in great detail for the bacterial CE15 members and, in this study, we examined the genomic surroundings of the *Df*CE15 enzymes and discovered that each is positioned in proximity to other putative metabolic enzymes (Supplemental Tables S2-4).

Despite belonging to the *Bacteroidota* phylum, no PULs including CE15 genes were found in the *D. fermentans* genome when searching in the PUL Database (Terrapon et al. 2018), and in fact, the species appears to lack larger PULs encoding more than three carbohydrate-active enzymes. Outside of defined PULs, of the three *D. fermentans* CE15 genes, the neighborhood surrounding *Df*CE15C stands out as the most likely to be geared towards glucuronoxylan catabolism, due to the presence of genes annotated as putative GH10 xylanases, a feruloyl esterase, and several sugar transporters mingled with several putative gene products lacking significant homology to characterized proteins. Interestingly, upstream lies another cluster of genes whose products are annotated for putative uronate metabolism. Within this larger region, transcriptional regulators as well as homologs of *susC* and *susD* (generally used as indicators of PULs) from *Bacteroides thetaiotaomicron* were also found, giving further support to the hypothesis that *Df*CE15C could be part of a large *D. fermentans* PUL, though transcriptomic analyses would be needed to verify this. *Df*CE15B is also found in a cluster encoding putative metabolic proteins; in this case with enzymes such as sugar kinases, phosphatases, sugar family transporters, and the CE15 gene is found beside a putative *myo*-inositol 1-phosphate synthase. In contrast, *Df*CE15A is not found in immediate proximity to homologs of proteins associated with any metabolic functions, though several of the genes lack homology to characterized proteins and cannot at this point be annotated.

### Characterization reveals distinct substrate specificities for *D. fermentans* CE15 enzymes

To gain deeper insight into the roles of the *Df*CE15 enzymes, each protein was recombinantly produced in *E. coli* and purified by immobilized metal ion affinity chromatography; the purification to homogeneity was assessed by SDS-PAGE. (Supplemental Fig. S1). All the enzymes were active on GlcA esters, and their hydrolytic activity on BnzGlcA was assessed at different pH values. Although the pH optima differed (broadly between pH 7.5 and 9.0 for *Df*CE15A, pH 7.5 for *Df*CE15B, and pH 8.5 for *Df*CE15C), all the enzymes had > 75% of maximal activity between pH values 7.5 and 8.5 (Supplemental Fig. S2). *Df*CE15A and *Df*CE15C maintained > 80% of their maximal activity at pH 9.5, but significant rates of auto-hydrolysis of the GlcA ester in alkaline conditions above this pH prevented an accurate assessment of the enzymatic contribution. Similar pH optimum ranges for different GEs originating from the same organism have been observed previously with other bacteria encoding multiple CE15 members (Arnling Bååth et al. 2018). The melting temperatures of the *D. fermentans* enzymes were between 40 and 50 °C (Supplemental Fig. S3). The kinetic parameters were determined for each enzyme using available model substrates; interestingly, this analysis revealed a distinct activity profile for each enzyme (Table 1). *Df*CE15C was extremely efficient at turning over the LCC mimic substrate BnzGlcA, with its catalytic efficiency being amongst the highest observed for a CE15 member to date (Arnling Bååth et al. 2019; Seveso et al. 2024). The enzyme had a similarly high catalytic efficiency for both the benzyl and methyl-substituted GlcA, a feature seen with some other bacterial CE15 members like *Ot*CE15A and *Su*CE15C (Arnling Bååth et al. 2018) but not the other two *D. fermentans* enzymes. *Df*CE15B, although having considerable activity towards BnzGlcA, had a ~ 10-fold decreased catalytic efficiency for MeGlcA, principally owing to a ~ 10-fold increase in *K*_M_. In contrast to both *Df*CE15B and *Df*CE15C, *Df*CE15A could not be saturated with either benzyl or methyl GlcA esters, and its catalytic efficiency, estimated by linear regression of activity over substrate concentration, was lower by 100- to 500-fold relative to the other enzymes, respectively. MeGalA was a poor substrate for all the *Df*CE15 enzymes with none of them able to be saturated up to 10 mM of the ester, revealing the enzymes to strictly discriminate between GlcA- versus GalA-based substrates, similar to studied fungal enzymes (Duranová et al. 2009; Larsbrink and Lo Leggio 2023). Given the proximity of *Df*CE15B to a putative *myo*-inositol 1-phosphate synthase in the genome, we tested if any of the *Df*CE15s could be inhibited by *myo*-inositol but did not find any significant inhibition of any of the enzymes catalyzing BnzGlcA hydrolysis (1 mM) with up to 2.5 mM added *myo*-inositol.

**Table 1:** Kinetics of the *Df*CE15 esterase activities. The *Df*CE15A-3v variant harbors the three mutations N142F, R143A, and G242L

Enzyme	Substrate	*K*_M_ (mM)	*k*_cat_ (s^−1^)	*k*_cat_/*K*_M_ (s^−1^ mM^−1^)
*Df*CE15A	BnzGlcA	Could not be saturated up to 50 mM		0.157 ± 0.0076
	MeGlcA	Could not be saturated up to 25 mM		0.351 ± 0.033
	MeGalA	Could not be saturated up to 50 mM		0.0120 ± 0.0017
*Df*CE15A-3v	BnzGlcA	23.5 ± 2.9	1.19 ± 0.055	0.0505 ± 0.0065
	MeGlcA	Could not be saturated up to 25 mM		0.0249 ± 0.0013
*Df*CE15B	BnzGlcA	2.97 ± 0.020	46.6 ± 0.056	15.7 ± 0.11
	MeGlcA	18.1 ± 1.5	31.6 ± 2.4	1.75 ± 0.20
	MeGalA	Could not be saturated up to 50 mM		0.571 ± 0.19
*Df*CE15B-Q318W	BnzGlcA	1.83 ± 0.055	46.2 ± 0.59	25.2 ± 0.82
	MeGlcA	5.93 ± 0.21	29.3 ± 0.59	4.94 ± 0.20
*Df*CE15C	BnzGlcA	1.00 ± 0.051	86.3 ± 2.1	86.0 ± 4.9
	MeGlcA	1.08 ± 0.14	85.6 ± 2.3	80.3 ± 13
	MeGalA	Could not be saturated up to 50 mM		0.545 ± 0.079

### Structural comparisons

**Fig. 2:**
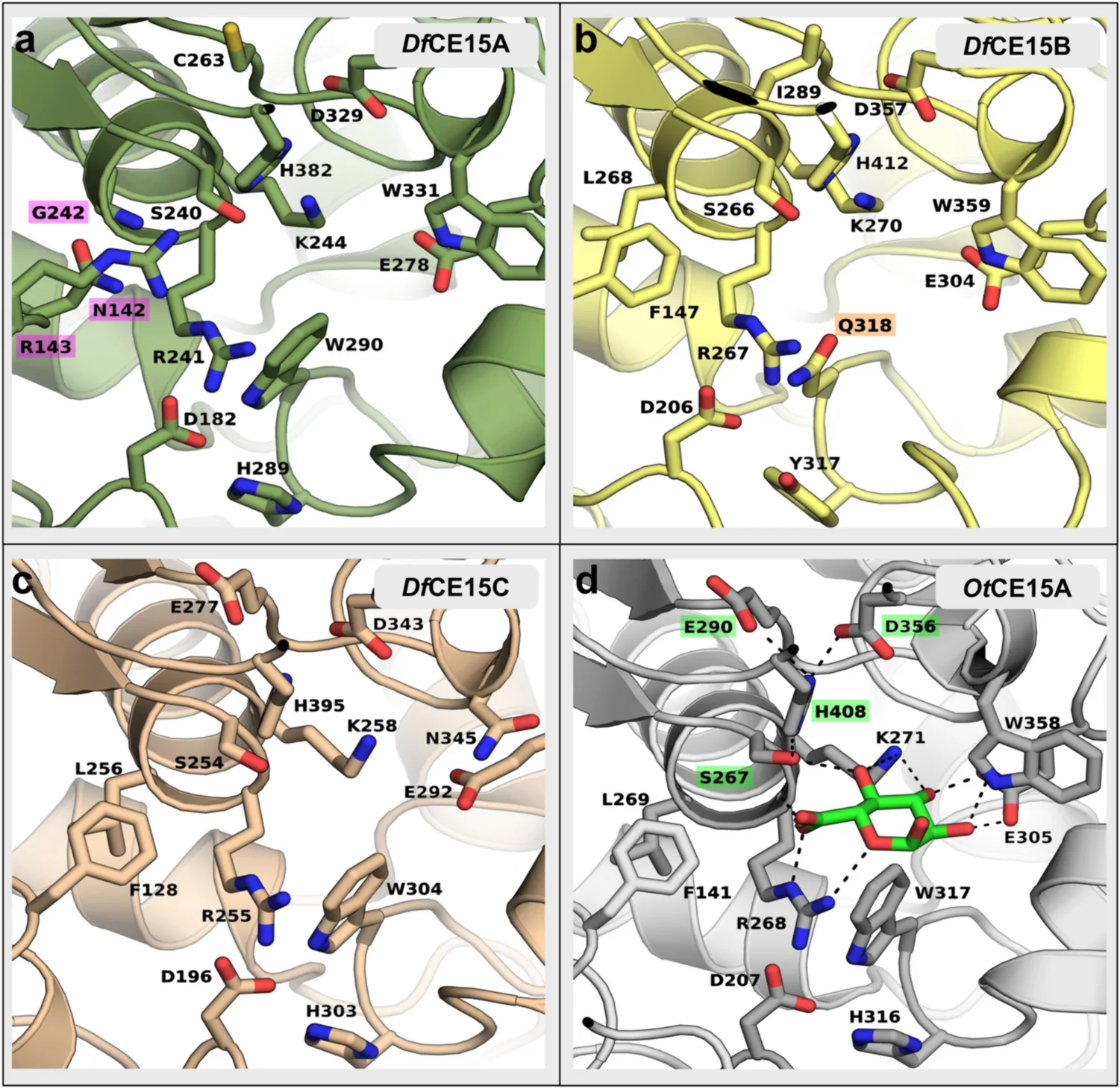
Comparison of CE15 protein structure models. The predicted models of the active site pocket of *Df*CE15A (**a**), *Df*CE15B (**b**), and *Df*CE15C (**c**) and the experimentally determined model of the active site pocket of *Ot*CE15A with the bound glucuronate molecule shown as green sticks (**d**; PDB accession: 6SYR). The catalytic triad residues in *Ot*CE15A are highlighted in green. The residues targeted for substitution in *Df*CE15A and *Df*CE15B are highlighted in magenta and orange, respectively. The figure was made using PyMOL 2.5

We sought to better understand the molecular determinants influencing the different activity levels observed in the *Df*CE15 enzymes, as this knowledge could also contribute to furthering our understanding of the diversity of activity levels across CE15. Given the availability of experimentally determined structures for CE15 members which provide valuable insights for predictive modeling, we decided to utilize the in silico predictions generated by AlphaFold2 (Jumper et al. 2021; Varadi et al. 2022), accessible through UniProt (Consortium 2023), as models for structural analysis. Excluding the signal peptide region, more than 98% of the constructed *Df*CE15 models had pLDDT confidence scores surpassing 90, indicating highly reliable predictions (Supplemental Fig. S4). Although only sharing between 25 and 45% sequence identity to other characterized CE15 members, as well as to each other (Supplemental Table S5), the models indicate considerable sequence conservation in the active site and substrate-binding pocket (Fig. 2). None of the *Df*CE15 enzymes contain additional modules, such as carbohydrate binding modules or glycoside hydrolase domains occasionally found with some glucuronoyl esterases.

Conserved in CE15 is the catalytic triad, composed of a serine, histidine, and an acidic residue(s) (Supplemental Fig. S5). The position of the acidic residue differs in the family, being either a glutamate at the canonical position (end of β7 within the ABH superfamily) or an aspartate at the noncanonical position (end of β6) (Arnling Bååth et al. 2018; Ernst et al. 2020). All fungal CE15 members structurally characterized to date have a glutamate at the noncanonical position while bacterial members have been observed with residues in either position, or in some cases both positions, such as *Ot*CE15A (Mazurkewich et al. 2019). All the three *D. fermentans* enzymes possess the expected catalytic serine and histidine residues; *Df*CE15A and *Df*CE15B have their putative catalytic acidic residues at the canonical ABH position, while in *Df*CE15C, putative acidic residues are found at both positions, similarly to *Ot*CE15A. Also, the conserved active site arginine, proposed to stabilize the transition state intermediate (Arnling Bååth et al. 2018; Mazurkewich et al. 2019; Zong et al. 2022), is conserved in all the three enzymes, collectively supporting catalytic competence. Beyond the catalytic residues, several residues making up the uronate binding pocket are similarly conserved or have residues of similar functionalities (Fig. 2). However, some amino acid residue differences between *Df*CE15A and *Df*CE15B relative to well-functioning CE15 members could be the root of the observed differences in activity.

### Investigations of differences in *Df*CE15 molecular activity determinants

Several bacterial CE15 members, such as *Df*CE15C, have a similar catalytic efficiency for both BnzGlcA and MeGlcA, indicating that GlcA is the principal moiety recognized by the enzyme. In contrast, *Df*CE15B has a catalytic efficiency for MeGlcA ~ 10-fold lower than BnzGlcA, principally resulting from a six-fold increase in *K*_M_. The predicted uronate binding clefts of *Df*CE15B and *Df*CE15C are quite similar with only a few differences observed and are both similar to *Ot*CE15A (Fig. 2, Supplemental Fig. S5). However, one notable difference observed in *Df*CE15B is the presence of glutamine residue (Gln318) found in place of a conserved tryptophan (W317 in *Ot*CE15A, Fig. 2) typically lining the bottom of the uronate binding pocket. Here, we hypothesized that this conserved tryptophan is required to support the correct positioning of the GlcA unit and that the substitution with a smaller glutamine in *Df*CE15B could cause the higher *K*_M_ for MeGlcA than BnzGlcA, where the larger latter substrate might compensate for the suboptimal uronate-binding cleft. Indeed, substitution of the glutamine with tryptophan led to a variant (*Df*CE15B-Q318W) with ~ 3-fold decrease in *K*_M_ for MeGlcA while only a 1.5-fold decrease in *K*_M_ for BnzGlcA, supporting the proposal that the bulky tryptophan side chain aids in properly positioning the GlcA moiety (Table 1). However, the *Df*CE15B-Q318W variant still had a fivefold difference in its catalytic efficiency for MeGlcA versus BnzGlcA indicating that additional determinants are contributing to this difference in the substrate recognition which is, at this moment, not obvious when comparing the predicted models. As shown in the alignment (Supplemental Fig. S5), the tryptophan residue is typically conserved in both fungal and bacterial CE15 enzymes, suggesting that substitutions in this position are not beneficial for the most common GE activity found in the family.

The activity of *Df*CE15A towards model substrates was, as mentioned, considerably diminished relative to competent GEs (Table 1). The protein has considerable sequence conservation in the proposed uronate-binding cleft indicating that differences elsewhere in the protein must lead to the observed differences (Fig. 2). A feature found amongst bacterial CE15 members characterized thus far is the presence of an inserted region (referred to as Region 2, Supplemental Fig. S6) which extends across and surrounds a cleft where the alcohol portion of the ester substrate, such as lignin fragments, is proposed to reside (Arnling Bååth et al. 2018, 2019). Although there is considerable diversity in sequence and structural features of these inserted regions, competent bacterial GEs, such as *Ot*CE15A and *Df*CE15C, contain a conserved phenylalanine residue (Phe141 in *Ot*CE15A, Phe128 in *Df*CE15C) located behind the catalytic serine (Fig. 2, Supplemental Fig. S5). This residue is situated at the beginning of Region 2, lining the bottom of the solvent exposed cleft, and is surrounded by variable hydrophilic residues which may support binding of partially hydrophilic aromatic substituents, such as lignin fragments (Arnling Bååth et al. 2018). The model of *Df*CE15A revealed a significantly smaller Region 2 than that found in most bacterial members characterized thus far, and it has two distinct differences in the cleft which could contribute to its poor activity. Firstly, *Df*CE15A lacks the conserved phenylalanine of Region 2, found instead as an asparagine (Asn142), and it additionally lacks a aliphatic residue, such as Leu269 in *Ot*CE15A and Leu256 in *Df*CE15C, which is often found to position the phenylalanine and is found instead as a glycine (Gly242) in *Df*CE15A. Secondly, the region in *Df*CE15A contains an additional arginine residue predicted to occupy the space above the conserved phenylalanine in the cleft which would then likely cause steric clashing with the alcohol moieties of the uronate esters. Thus, we hypothesized that modification of the protein by enlarging this space of the cleft, by way of substituting the large side chain of Arg143 with the small side chain of alanine (R143A) and by restoring the conserved phenylalanine and its positioning (N142F and G242L), would lead to an enzyme much more competent towards the model substrates. A variant with the combined N142F/R143A/G242L substitutions, herein referred to as *Df*CE15A-3v, was thus constructed. This variant could successfully be saturated with BnzGlcA, albeit with a *K*_M_ ~ 20-fold greater than that of *Df*CE15C and also maintained a low catalytic efficiency for the model substrate (Table 1). The enzyme was not able to be saturated with MeGlcA indicating that while the residue substitutions aided the enzyme, additional and not easily identifiable determinants further hindered activity on GlcA substrates.

## Discussion

CE15 members are encoded by a range of microorganisms from diverse ecological niches, from tropical (Hüttner et al. 2017) and temperate (Arnling Bååth et al. 2018) soils to the guts of various animals including termites (Marynowska et al. 2017) and humans (Kmezik et al. 2021). GE activity is the principal activity found in the family with members shown to cleave ester bonds between xylan and lignin alcohols in lignocellulosic LCCs (Arnling Bååth et al. 2016; d'Errico et al. 2016; Mosbech et al. 2019; Raji et al. 2021; Zong et al. 2022). However, since many microorganisms encode multiple CE15 enzymes with a diversity of activities on synthetic and natural substrates (Arnling Bååth et al. 2018, 2019; Hüttner et al. 2017), it is possible that they fulfil different roles, such as targeting different forms of LCCs, which would not be unexpected given the rather unpredictable structure of lignin. Moreover, the fact that some CE15 genes are found in PULs or genetic neighborhoods not appearing to target xylan further suggests that other activities than xylan deconstruction could be found within the family (Arnling Bååth et al. 2018; Seveso et al. 2024). The three CE15 enzymes from *D. fermentans* studied in this work served as a model to begin to explore additional potential differences in the family as each is quite distinct and especially two reside in unexplored clades of the CE15 phylogenetic tree (Fig. 1).

Considerable diversity exists within CE15, manifesting in variations in enzyme activity and specificities amongst its members. Notably, bacterial CE15 members, differently from their fungal counterparts (d'Errico et al. 2015; Duranová et al. 2009), exhibit a distinctive capability to efficiently utilize GlcA esters without methylation of the 4-hydroxyl group (Arnling Bååth et al. 2018). The canonical esterase mechanism, using the catalytic core Ser-His-Acid, is a reaction that exhibits the formation/release of the acyl enzyme, and either step could be rate-limiting depending on the specific enzyme (Rauwerdink and Kazlauskas 2015). Amongst bacterial GEs, several individual enzymes, like *Ot*CE15A (Arnling Bååth et al. 2018; Mazurkewich et al. 2019), appear indiscriminate towards the alcohol substituent of the ester and exhibit similar turnover rates for GlcA ester substrates regardless of the alcohol moiety, which is consistent with deacylation as the rate-determining step for these enzymes (Zong et al. 2022). However, for some GEs, the nature of the alcohol substituent can influence the *K*_M_ parameters with some having significant increases in *K*_M_ for MeGlcA, up to 10-fold, compared to the larger BnzGlcA (Arnling Bååth et al. 2018, 2019). Here, we demonstrated that modifying the carbohydrate binding cleft in *Df*CE15B with the Q318W variant, substituting the rare occurrence of a glutamine residue in the position of the conserved tryptophan residue at the bottom of the uronate binding pocket, resulted in a threefold decrease in *K*_M_ for MeGlcA compared to a 1.5-fold decrease in *K*_M_ for BnzGlcA. This indicates that the substitution contributed to improved affinity for the GlcA unit which was particularly notable for the smaller MeGlcA substrate. Moreover, the relatively unchanged *K*_M_ for BnzGlcA with the Q318W substitution supports the notion that determinants outside the carbohydrate cleft play a role in defining affinity and specificity for various alcohol substituents in at least some CE15 members, and these contributions are sufficient in the wild-type *Df*CE15B to overcome the sub-optimal carbohydrate binding cleft for larger alcohol substituents. A few aromatic residues are found in Region 2 of *Df*CE15B which could aid in driving the specificity of the enzyme towards the larger BnzGlcA (Supplemental Fig. S5). However, the confidence of the model prediction in Region 2 of *Df*CE15B, and the other *Df*CE15 enzymes, is not as high as the rest of the protein, likely owing to the heterogeneity observed in this region amongst structurally determined members, making predictions for roles of residues in this area highly speculative but also warrants further study.

The in silico analysis performed on the *Df*CE15 enzymes revealed high sequence conservation in the binding cleft with the conserved catalytic triad (two acidic residues for *Df*CE15C, like *Ot*CE15A (Arnling Bååth et al. 2018)) and the oxyanion-stabilizing arginine (Arnling Bååth et al. 2018; Zong et al. 2022) (Fig. 2, Supplemental Fig. S5). The most substantial difference observed amongst the proteins is the significantly smaller Region 2 in *Df*CE15A, here predicted to result in a more open cleft than is typical of bacterial GEs. *Df*CE15A has poor activity and could not be saturated with synthetic GE substrates. Despite significant variability in sequence identity within Region 2 across the family, a similar cleft is formed by the presence of loops which house a highly conserved phenylalanine and leucine residues (Phe141 and Leu269 in *Ot*CE15A) which have been proposed to contribute to substrate accommodation and specificity (Arnling Bååth et al. 2018, 2019; De Santi et al. 2017; Mazurkewich et al. 2019). The introduction of phenylalanine and leucine into analogous positions in *Df*CE15A, originally occupied by Asn142 and Gly242, respectively, resulted in a saturable enzyme. However, there was no observed improvement in the poor catalytic efficiency for the enzyme. This suggests that additional determinants, likely located in Region 2, contribute to the enzyme’s lack of activity with synthetic GE substrates. Whether this is tied to the enzyme having become crippled through unproductive substitutions or that it was here not assayed on a substrate closer to its natural target remains unclear. The role(s) Region 2 play in affecting activity and specificity in CE15 members has not been explored, and future studies coupling substrate specificities to the breadth of diversity in this region would greatly aid in advancing our understanding of the biological roles these enzymes play.

*D. fermentans* belongs to the genetically diverse *Cytophagaceae* family, which is not well-studied. The *Df*CE15 genes were not found in obvious and predicted PULs or similar regions that could strongly indicate their function, apart from the region around *Df*CE15C, which contains putative xylanases and a feruloyl esterase, and also SusC/D-like proteins, suggesting a putative PUL or PUL-like system for (arabino-)glucuronoxylan deconstruction. *Z. mays*, with its complex xylan structure, would require a diverse set of enzymes for degradation, and potentially, *Df*CE15C is part of a large PUL geared for this (Chelius and Triplett 2000; Rogowski et al. 2015). However, evaluation of the expression of all putative PUL genes when the organism is grown on different xylans would be needed to confirm this supposition. The high efficiency of *Df*CE15C on the LCC mimic BnzGlcA does point towards a role in xylan-lignin LCC cleavage. *Df*CE15B’s catalytic efficiency with GlcA substrates supports its classification as a GE; furthermore, since it was found next to a putative *myo*-inositol 1-phosphate synthase in the genome, we assessed its interaction with *myo*-inositol, as *myo*-inositol esters have previously been identified in *Z. mays* (Chisnell 1984). No strong interactions could however be observed, and again, a potential other angle to find connections amongst the CE15 genes and others closely positioned in the genome would be larger transcriptomics experiments. *Df*CE15A exhibited a very poor performance on the synthetic GE substrates, and it belongs to a phylogenetic clade significantly distant from previously characterized CE15 members. While its function in the metabolism of *D. fermentans* still remains unclear, it represents a model system to define/explore the determinants modulating GE activity. In summary, this study contributes to our understanding of GEs and CE15 as a whole, specifically shedding light on the intricate details regarding the relationship between structure and function within the binding pocket and active site of GEs, aiding our understanding of the diversity that exists within the protein family.

## Supplementary information

Below is the link to the electronic supplementary material.

Supplementary file1 (PDF 2765 KB)

## Data Availability

All data generated in this study are described in the published article and its additional Supplementary material. Raw data may be provided upon reasonable request from the corresponding authors.
