# Supplementary material for: Exploration of three Dyadobacter fermentans enzymes uncovers molecular activity determinants in CE15
Source: Appl Microbiol Biotechnol. 2024 May 15;108:335. doi: 10.1007/s00253-024-13175-6 (PMC11096219; doi:10.1007/s00253-024-13175-6)
Supplement: Supplementary file 1 [file 253_2024_13175_MOESM1_ESM.pdf]

Applied Microbiology and Biotechnology

Supplemental information for:

Exploration of three *Dyadobacter fermentans* enzymes uncovers molecular activity determinants in CE15

Miriam Carbonaro[1*], Scott Mazurkewich[2*#], Gabriella Fiorentino[1], Leila Lo Leggio[3], Johan Larsbrink[2#]

[1]Department of Biology, University of Naples Federico II, 80126 Naples, Italy.

[2]Wallenberg Wood Science Center, Division of Industrial Biotechnology, Department of Life Sciences, Chalmers University of Technology, SE-412 96 Gothenburg, Sweden.

[3]Department of Chemistry, University of Copenhagen, Universitetsparken 5, DK-2100 Copenhagen, Denmark

[*]These authors contributed equally

[#]Correspondence: scott.mazurkewich@chalmers.se and johan.larsbrink@chalmers.se

Content: Supplementary tables S1-S5, Supplementary figures S1-S6.

Supplementary tables

Table S1. Primers used for the cloning of *D. fermentans* CE15 genes, and for the mutated variants of *Df*CE15A and *Df*CE15B.

Gene	Primer	5'-3' sequence
*Df*CE15A	*Df*CE15Af	CTTCCAGGGCCATAGTCAGAATTATGATGAATCCAAAGTGCCGC
	*Df*CE15Ar	TGGTGGTGCTCGAGTCTAAAATTGTTTAGCGAAACGGATGAATTTATC
*Df*CE15B	*Df*CE15Bf	CTTCCAGGGCCATAGTCAACAAGCCGCGAATCGACCG
	*Df*CE15Br	TGGTGGTGCTCGAGTCTACAAATACTTTTGCATGTAGCGTATGAAGAG
*Df*CE15C	*Df*CE15Cf	CTTCCAGGGCCATAGTCAAGATGTGCAGCGACTACCCG
	*Df*CE15Cr	TGGTGGTGCTCGAGTCTATTGCAAATGTAAATCCGAGAAGTTGAGAAT
*Df*CE15A-3v	*Df*CE15A-N142F/R143Af	ATCAATTTCGCTGGGAAGGAC
	*Df*CE15A-N142F/R143Ar	CTTCCCAGCGAAATTGATCAG
	*Df*CE15A-G242Lf	TCGCGCCTTGGTAAAACTTC
	*Df*CE15A-G242Lr	TTTACCAAGGCGCGAATGCC
*Df*CE15B-Q318W	*Df*CE15B-Q318Wf	TTGTATTGGTTTGCGCCCAAT
	*Df*CE15B-Q318Wr	CGCAAACCAATACAAGTACCG

Table S2. Genomic neighbourhood of *Df*CE15A. The closest characterized homolog for a neighbouring gene was identified through either primary sequence analysis using BLAST (Altschul et al. 1990) or structural analysis through Foldseek (van Kempen et al. 2023) from the AlphaFold2 (Varadi et al. 2022) predicted structure found on Uniprot (Consortium 2023). *Df*CE15A is highlighted in green and sequences of a cluster of 3 or more putatively annotated genes associated with a specific function are additionally highlighted.

Start	End	Direction	New locus tag	Old locus tag	NCBI Accession	Closest characterized homolog	% Seq Identity	% Query coverage	Citation	Name or Putative annotation	Putative cluster annotation
3686925	3687737	-	*DF*ER_RS14895	*Dfer*_3014	WP_015812478.1	LpxA a UDP-*N*-acetylglucosamine acyltransferase from *Bacteroides fragilis* 9343	49	94	PMID: 25945572	UDP-GlcNAc acyltransferase	
3687734	3689125	-	*DF*ER_RS14900	*Dfer*_3015	WP_015812479.1	Dual domain - LpxC - UDP-(3-O-((R)-3-hydroxymyristoyl))-*N*-acetylglucosamine deacetylase from *Yersinia enterocolitica* & acyl-carrier-protein] dehydratase FabZ from *E. coli*	38	63	PMID: 21171638	CE11 - Deacetylase & Dehydratase carrier protein	
3689174	3690214	-	*DF*ER_RS14905	*Dfer*_3016	WP_015812480.1	*N*-Acyltransferase of lipid A biosynthesis	37	97	PMID: 17360522	UDP-*N*-acyltransferase	
3690296	3691528	-	*DF*ER_RS14910	*Dfer*_3017	WP_229206007.1	Phosphohydrolase (BT4208) from *Bacteroides thetaiotaomicron* VPI-5482	51	98	Unpublished	dNTPase	
3691649	3691722	+	*DF*ER_RS14915	*Dfer*_R0009	tRNA	tRNA	-	-	-	tRNA	
3691822	3692013	+	*DF*ER_RS14920	*Dfer*_3018	WP_015812482.1	Cold shock protein E from *Salmonella typhimurium*	49	96	PMID: 20054119	Cold shock protein	
3692138	3693040	+	*DF*ER_RS14925	*Dfer*_3019	WP_015812483.1	Predicted lipid A biosynthesis acyltransferase	-	-	-	Lipid A biosynthesis acyltransferase	
3693050	3694216	-	*DF*ER_RS14930	*Dfer*_3020	WP_015812484.1	Sialidase from *Micromonospora viridifaciens*	33	88	PMID: 8591030	GH33 (sialidase?)	Possible lipid A biosynthesis & modification cluster
3694227	3695732	-	*DF*ER_RS14935	*Dfer*_3021	WP_015812485.1	*Xanthomonas* xyloglucan acetylesterase	34	92	https://doi.org/10.1038/s41467-021-24277-4	CE20 (sialic acid acetyl est?)	
3695729	3698380	-	*DF*ER_RS14940	*Dfer*_3022	WP_015812486.1	Sialic acid symporter from *Proteus mirabilis* with a seperate kelch-type beta propeller domain	37	54	PMID: 29717135	Sugar transporter	
3698380	3699321	-	*DF*ER_RS14945	*Dfer*_3023	WP_015812487.1	*N*-acetylneuraminate lyase from *Aliivibrio salmonicida*	38	90	PMID: 31185017	*N*-acetylneuraminate lyase - class I aldolase	
3699352	3700926	-	*DF*ER_RS14950	*Dfer*_3024	WP_015812488.1	SusD homolog from *Bacteroides fragilis* NCTC 9343	10	90	NA	SusC/D - Sugar binding TonB receptor	
3700931	3704317	-	*DF*ER_RS14955	*Dfer*_3025	WP_015812489.1	SusC homolog from *Bacteroides thetaiotaomicron* VPI-5482	27	90	PMID: 28077872	SusC/D - Sugar binding TonB receptor	
3704501	3705202	+	*DF*ER_RS14960	*Dfer*_3026	WP_015812490.1	Uxu operon regulator from *Haemophilus influenzae* Rd KW20 - FadR/GntR type	32	83	P44487.1	Transcriptional regulator	
3705243	3705932	-	*DF*ER_RS14965	*Dfer*_3027	WP_229206008.1	2-Hydroxy-6-oxo-6-phenylhexa-2,4-dienoate hydrolase (BphD homolog) from *Rhodococcus jostii* RHA1	23	88	https://doi.org/10.2183/pjab.73.154	BphD type hydrolase - alpha/beta-hydrolase	
3706236	3706931	+	*DF*ER_RS14970	*Dfer*_3028	WP_187293386.1	Predicted bacterial Ig-like domain containing protein	-	-	-	-	
3706966	3707937	-	*DF*ER_RS14975	*Dfer*_3029	WP_015812493.1	Predicted peptidase S74 containing domain	-	-	-	-	
3708192	3708371	-	*DF*ER_RS29920	*Dfer*_3030	WP_015812494.1	None - unknown (59 aa)	-	-	-	-	
3708611	3709012	+	*DF*ER_RS14990	*Dfer*_3031	WP_015812495.1	None - unknown (133 aa)	-	-	-	-	
3709009	3709236	-	*DF*ER_RS14995	*Dfer*_3032	WP_015812496.1	None - unknown (75 aa)	-	-	-	-	
3709360	3710535	-	*DF*ER_RS15000	*Dfer*_3033	WP_229206009.1	Glucuronoyl esterase from *Opitutus terrae Ot*CE15A	39	94	PMID: 30083226	*Df*CE15A	
3710721	3711095	+	*DF*ER_RS15005	*Dfer*_3034	WP_041735155.1	None - unknown (124 aa)	-	-	-	-	
3711190	3711723	+	*DF*ER_RS15010	*Dfer*_3035	WP_015812499.1	RNA polymerase sigma factor SigM *Mycobacterium tuberculosis*	25	81	H8F2P4.1		
3711723	3713324	+	*DF*ER_RS15015	*Dfer*_3036	WP_015812500.1	Predicted Outer membrane protein beta-barrel domain-containing protein	-	-	-	-	
3713349	3715559	-	*DF*ER_RS15020	*Dfer*_3037	WP_015812501.1	SusC homolog from *Bacteroides thetaiotaomicron* VPI-5482	10	90	PMID: 28077872	SusC/D - sugar binding TonB receptor	
3715889	3716446	-	*DF*ER_RS15025	*Dfer*_3038	WP_041735158.1	None - unknown (169 aa)	-	-	-	-	
3716623	3716961	+	*DF*ER_RS15030	*Dfer*_3039	WP_015812503.1	None - Cupin domain (112 aa)	-	-	-	-	
3716986	3717729	-	*DF*ER_RS15035	*Dfer*_3040	WP_015812504.1	Short-chain dehydrogenase/reductase FabG from *Bacillus sp.*	42	98	PMID: 24212572	Dehydrogenase	
3717952	3719268	+	*DF*ER_RS15040	*Dfer*_3041	WP_041735160.1	GH158 - endo-laminarinase from beta(1,3)-glucan utilization locus of *Bacteroides uniformis*	26	91	PMID: 32265336	GH	
3719265	3722207	+	*DF*ER_RS15045	*Dfer*_3042	WP_015812506.1	GT2 - cellulose synthase from *Cereibacter sphaeroides* + a GH domain similar to GH158	30	28	PMID: 26958837	GT + GH	Sugar metabolism
3722201	3723877	-	*DF*ER_RS15050	*Dfer*_3043	WP_015812507.1	Predicted 2 domains: Nt= ??? (probably another GH) & Ct = GH26 (beta-mannanase)	-	20	PMID: 15987675	??? + GH	
3724066	3724398	-	*DF*ER_RS15055	*Dfer*_3044	WP_015812508.1	None - unknown (110 aa)	-	-	-	-	
3724610	3725785	-	*DF*ER_RS15060	*Dfer*_3045	WP_015812509.1	Glutathionylspermidine synthetase/amidase from *Escherichia coli* K-12	37	91	PMID: 36745518	Ligase	
3725832	3726443	-	*DF*ER_RS15065	*Dfer*_3046	WP_015812510.1	None - unknown (203 aa)	-	-	-	-	
3726626	3727912	-	*DF*ER_RS15070	*Dfer*_3047	WP_015812511.1	Aminotransferase PvdN from *Pseudomonas aeruginosa*	24	98	PMID: 27139833	Transferase/esterase	
3727984	3728742	-	*DF*ER_RS15075	*Dfer*_3048	WP_015812512.1	Lysostaphin-type metalloendopeptidase from *S. aureus*	30	34	PMID: 16269153	Peptidase/esterase	
3728904	3729875	+	*DF*ER_RS15080	*Dfer*_3049	WP_015812513.1	4-Hydroxy-2,3-trans-nonenal reductase from *Bacillus halodurans* (or 1-deoxyxylulose-5-phosphate synthase)	30	95	PMID: 16242712	Oxidoreductase	
3730103	3731326	-	*DF*ER_RS15085	*Dfer*_3050	WP_015810681.1	Putative transposase y4rJ from *Sinorhizobium fredii* NGR234	28	65	P55643.1	Transposase	
3731421	3731831	-	*DF*ER_RS15090	*Dfer*_3051	WP_015812514.1	None - unknown (136 aa)	-	-	-	-	
3732017	3734278	+	*DF*ER_RS15095	*Dfer*_3052	WP_015812515.1	SusC homolog from *Bacteroides thetaiotaomicron* VPI-5482	12	90	PMID: 28077872	SusC/D - sugar binding TonB receptor	
3734425	3734733	+	*DF*ER_RS15100	*Dfer*_3053	WP_015812516.1	CBM20-like from a GH13 Rv1326c of *Mycobacterium tuberculosis*	67	20	PMID: 20444687	Sugar binding/acting	Sugar metabolism
3734865	3735785	-	*DF*ER_RS15105	*Dfer*_3054	WP_015812517.1	3-Keto-α-D-gulosides epimerase from *Escherichia coli* K-12 MG1655	16	95	PMID: 19255464	Sugar epimerase	
3735867	3738569	-	*DF*ER_RS15110	*Dfer*_3055	WP_229206010.1	PQQ-dependent glucose dehydrogenase from *Acinetobacter calcoaceticus*	14	50	no ref	Sugar dehydrogenase	

Table S3. Genomic neighbourhood of *Df*CE15B. The closest characterized homolog for a neighbouring gene was identified through either primary sequence analysis using BLAST (Altschul et al. 1990) or structural analysis through Foldseek (van Kempen et al. 2023) from the AlphaFold2 (Varadi et al. 2022) predicted structure found on Uniprot (Consortium 2023). *Df*CE15B is highlighted in green and sequences of a cluster of 3 or more putatively annotated genes associated with a specific function are additionally highlighted.

Start	End	Direction	New locus tag	Old locus tag	NCBI Accession	Closest characterized homolog	% Seq Identity	% Query coverage	Citation	Name or Putative annotation	Putative cluster annotation
3801959	3802300	+	*DF*ER_RS15400	*Dfer*_3112	WP_015812575.1	Metallochaperone *Staphylococcus aureus*	24	50	PDB: 6FF2	Metal chaperone	
3802319	3803716	+	*DF*ER_RS15405	*Dfer*_3113	WP_015812576.1	OmpF transporter from *E. coli*	9	80	PMID: 29038444	Transporter	
3803767	3804510	+	*DF*ER_RS15410	*Dfer*_3114	WP_015812577.1	Methyltransferase from *Streptomyces caelestis*	17	90	PMID: 24699640	Methyltransferase	
3804511	3804969	-	*DF*ER_RS15415	*Dfer*_3115	WP_015812578.1	Predicted transporter - DUF6691	-	-	-	Transporter	
3805072	3805647	-	*DF*ER_RS15420	*Dfer*_3116	WP_015812579.1	Predicted sulfur transporter	-	-	-	Transporter	
3806056	3806265	-	*DF*ER_RS15430	*Dfer*_3118	WP_015812580.1	None - unknown (69 aa)	-	-	-	-	
3806314	3807705	-	*DF*ER_RS15435	*Dfer*_3119	WP_015812581.1	Protein with beta-lactamase and rhodanese domains from *Alicyclobacillus acidocaldarius*	35	98	PDB: 3TP9	Metallo hydrolase	
3807930	3809315	-	*DF*ER_RS15440	*Dfer*_3120	WP_015812582.1	Multidrug and Toxin Compound Extrusion (MATE) transporter from *Vibrio cholerae* O1	22	86	PMID: 20861838	Transporter	
3809655	3810890	+	*DF*ER_RS15445	*Dfer*_3121	WP_015812583.1	GTPase HflX from *Escherichia coli*	40	87	PMID: 26458047	GTPase	
3810959	3811030	+	*DF*ER_RS15450	*Dfer*_R0010	tRNA	tRNA	-	-	-	-	
3811321	3812429	+	*DF*ER_RS15460	*Dfer*_3123	WP_222837289.1	Transposase from *E. coli*	12	90	PMID: 35654042	Transposase	
3812599	3813690	+	*DF*ER_RS15465	*Dfer*_3124	WP_015812584.1	None - unknown (363 aa)	-	-	-	-	
3813891	3814100	+	*DF*ER_RS15470	*Dfer*_3125	WP_015812585.1	None - unknown (69 aa)	-	-	-	-	
3814185	3815477	+	*DF*ER_RS15475	*Dfer*_3126	WP_015812586.1	Glucuronoyl esterase from *Opitutus terrae Ot*CE15A	36	100	PMID: 30083226	*Df*CE15B	
3815760	3816980	+	*DF*ER_RS15480	*Dfer*_3127	WP_015812587.1	Myo-inositol 1-phosphate synthase from *Mycobacterium tuberculosis*	42	89	PMID: 12005437	Inositol 1-phosphate synthase	
3817008	3817775	-	*DF*ER_RS15485	*Dfer*_3128	WP_015812588.1	Predicted DeoR type regulator	-	-	-	Transcription regulator	
3818116	3819381	+	*DF*ER_RS15490	*Dfer*_3129	WP_015812589.1	Predicted MFS transporter	-	-	-	MFS Transporter	
3819447	3820451	+	*DF*ER_RS15495	*Dfer*_3130	WP_015812590.1	Human 3-phosphoglycerate dehydrogenase	31	90	PMID: 29262655	2-hydroxy acid dehydrogenase	
3820463	3821908	+	*DF*ER_RS15500	*Dfer*_3131	WP_015812591.1	L-fuculose kinase from *Streptococcus Pneumoniae*	25	67	PMID: 24333485	Sugar kinase	
3821942	3823399	+	*DF*ER_RS15505	*Dfer*_3132	WP_015812592.1	Sugar kinases from *Synechococcus elongatus* PCC7942	30	87	PMID: 27223615	Sugar kinase	Sugar metabolism
3823402	3824004	+	*DF*ER_RS15510	*Dfer*_3133	WP_015812593.1	Phosphoserine phosphatase from *Hydrogenobacter thermophilus* TK-6	32	98	PMID: 23479726	Phosphatase/Phosphoglycerate mutase	
3824018	3824956	+	*DF*ER_RS15515	*Dfer*_3134	WP_015812594.1	Glycerophosphodiester phosphodiesterase from *Parabacteroides distasonis* ATCC 8503	50	89	PDB: 3MZ2	Phosphodiesterase	
3825141	3825494	+	*DF*ER_RS15520	*Dfer*_3135	WP_015812595.1	Predicted PadR type transcriptional regulator	-	-	-	Transcription regulator	
3825498	3825998	+	*DF*ER_RS30555		WP_229206013.1	Predicted ABC transporter associated protein	-	-	-	Transporter	
3825898	3828159	+	*DF*ER_RS15525	*Dfer*_3136	WP_229206014.1	Predicted ABC transporter	-	-	-	Transporter	
3828309	3829505	+	*DF*ER_RS15530	*Dfer*_3137	WP_015812596.1	L, D-transpeptidase from *E. coli*	37	56	PMID: 31015395	L, D-transpeptidase	
3829478	3830245	-	*DF*ER_RS15535	*Dfer*_3138	WP_015812597.1	Predicted murein L, D-transpeptidase	-	-	-	L, D-transpeptidase	
3830388	3831245	-	*DF*ER_RS15540	*Dfer*_3139	WP_015812598.1	Prephenate dehydrogenase from *Aquifex aeolicus*	38	88	PMID: 19279014	Dehydrogenase	
3831274	3832431	-	*DF*ER_RS15545	*Dfer*_3140	WP_015812599.1	Pyridoxal 5-phosphate (PLP)-dependent stereospecific transaminase from *Bacillus subtilis*	38	95	PMID: 32134000	Transaminase	

Table S4. Genomic neighbourhood of *Df*CE15C. The closest characterized homolog for a neighbouring gene was identified through either primary sequence analysis using BLAST (Altschul et al. 1990) or structural analysis through Foldseek (van Kempen et al. 2023) from the AlphaFold2 (Varadi et al. 2022) predicted structure found on Uniprot (Consortium 2023). *Df*CE15C is highlighted in green and sequences of a cluster of 3 or more putatively annotated genes associated with a specific function are additionally highlighted.

Start	End	Direction	New locus tag	Old locus tag	NCBI Accession	Closest characterized homolog	% Seq Identity	% Query coverage	Citation	Name or Putative annotation	Putative cluster annotation
6841666	6841789	-	*DFER_RS28420*	*Dfer_5694*	WP_015815123.1	2-Keto-myo-inositol dehydratase from *Clostridium tetani* E88	55	99	Q898E6.1	Sugar dehydratase	Possible uronate to amino acid synthesis cluster
6841789	6843654	-	*DFER_RS28425*	*Dfer_5695*	WP_015815124.1	Acetohydroxyacid synthase from *Arabidopsis thaliana*	27	77	PMID: 35690625	Acetohydroxyacid synthase	
6843693	6844634	-	*DFER_RS28430*	*Dfer_5696*	WP_015815125.1	Fructose-1,6-bisphosphate (FBP) aldolase from *Thermus aquaticus*	42	98	PMID: 14699122	Class II aldolase	
6844643	6846253	-	*DFER_RS28435*	*Dfer_5697*	WP_015815126.1	Sodium/galactose transporter from *Vibrio parahaemolyticus*	48	99	PMID: 21131949	Sugar transporter	
6846421	6847449	-	*DFER_RS28440*	*Dfer_5698*	WP_015815127.1	Myo-inositol 2-dehydrogenase from *Thermotoga maritima* MSB8	51	97	Q9WYP5.1	Sugar dehydrogenase	
6847477	6848943	-	*DFER_RS28445*	*Dfer_5699*	WP_015815128.1	Methylmalonate semialdehyde dehydrogenase from *Pseudomonas sp.* AAC	41	98	PMID: 28045390	Sugar dehydrogenase	
6848957	6849964	-	*DFER_RS28450*	*Dfer_5700*	WP_015815129.1	Fructokinase from *Halothermothrix orenii* H 168	25	97	PMID: 20493950	Sugar kinase	
6850215	6851078	-	*DFER_RS28455*	*Dfer_5701*	WP_015815130.1	Uncharacterized 5-deoxy-glucuronate isomerase from *Geobacillus kaustophilus* HTA426	39	88	Q5KYR2.1	KduI/IolB uronate isomerase	
6851205	6852236	+	*DFER_RS28460*	*Dfer_5702*	WP_015815131.1	Catabolite control protein A (CcpA) from *Bacillus megaterium*	32	97	PMID: 17500051	Transcription regulator	Sugar metabolism
6852350	6854977	+	*DFER_RS28465*	*Dfer_5703*	WP_143828846.1	None - unknown (875 aa)	-	-	-	-	
6855047	6856741	-	*DFER_RS28470*	*Dfer_5704*	WP_015815133.1	SusD homolog from *Bacteroides thetaiotaomicron* VPI-5482	26	94	PMID: 28077872	SusC/D - sugar binding TonB-dependent receptor	
6856752	6859787	-	*DFER_RS28475*	*Dfer_5705*	WP_015815134.1	SusC homolog from *Bacteroides thetaiotaomicron* VPI-5482	31	90	PMID: 28077872	SusC/D - sugar binding TonB-dependent receptor	
6860250	6860483	-	*DFER_RS28480*	*Dfer_5706*	WP_015815135.1	None - short sequence (74 aa)	-	-	-	-	
6860487	6860951	-	*DFER_RS28485*	*Dfer_5707*	WP_015815136.1	None - unknown (154 aa)	-	-	-	-	
6860996	6863305	-	*DFER_RS28490*	*Dfer_5708*	WP_310586751.1	ABC transporter from *E. coli* K-12 - uncharacterized	27	95	P77504.1	ABC Transporter	
6863315	6863539	-	*DFER_RS30800*		WP_310586752.1	None - short sequence (74 aa)	-	-	-	-	
6863553	6864227	-	*DFER_RS28495*	*Dfer_5709*	WP_015815137.1	ABC transporter from *Streptococcus pneumoniae* R6	49	98	PMID: 29335499	ABC Transporter	
6864404	6865099	+	*DFER_RS28500*	*Dfer_5710*	WP_041735644.1	Lysophospholipase A (TesA) from *Pseudomonas aeruginosa*	42	80	PMID: 23874889	Esterase	
6865135	6865686	-	*DFER_RS28505*	*Dfer_5711*	WP_015815139.1	None - unknown (183 aa) - DUF456 protein	-	-	-	-	
6865762	6865926	-	*DFER_RS29990*	*Dfer_5712*	WP_015815140.1	None - short sequence (54 aa) - DUF5670 protein	-	-	-	-	
6866152	6866970	+	*DFER_RS28510*	*Dfer_5713*	WP_015815141.1	Feruloyl esterase Fae1A from *Bacteroides intestinalis*	27	92	PMID: 28669823	Feruloyl esterase	
6866995	6868242	+	*DFER_RS28515*	*Dfer_5714*	WP_015815142.1	Glucuronoyl esterase from *Opitutus terrae Ot*CE15A	45	93	PMID: 30083226	*Df*CE15C	
6868278	6869531	+	*DFER_RS28520*	*Dfer_5715*	WP_015815143.1	L-fucose-proton symporter from *Escherichia coli* K-12	38	93	PMID: 20877283	MFS transporter	
6869843	6870742	+	*DFER_RS28525*	*Dfer_5716*	WP_015815144.1	GlcNAc kinase from *Plesiomonas shigelloides*	35	98	10.1101/2021.09.30.462564	Sugar kinase	
6870891	6872087	+	*DFER_RS28530*	*Dfer_5717*	WP_015815145.1	None - unknown (398 aa) - COG3503	-	-	-	-	
6872118	6873545	+	*DFER_RS28535*	*Dfer_5718*	WP_015815146.1	None - alpha/beta hydrolase fold predicted (2 domains predicted, other is beta-sandwich)	-	-	-	Esterase (likely AcEst of Fae)	
6873549	6873965	-	*DFER_RS28540*	*Dfer_5719*	WP_015815147.1	None - unknown (138 aa)	-	-	-	-	
6873987	6874511	-	*DFER_RS30650*	*Dfer_5720*	WP_015815148.1	None - unknown (174 aa)	-	-	-	-	
6874465	6876093	-	*DFER_RS28545*	*Dfer_5721*	WP_229206131.1	Predicted to have GH10-like fold	-	-	-	GH10	
6876210	6877340	+	*DFER_RS28550*	*Dfer_5722*	WP_041735648.1	Predicted to have GH10-like fold	-	-	-	GH10	
6877356	6878492	-	*DFER_RS28555*	*Dfer_5723*	WP_015815151.1	Uncharacterized MFS-type transporter from *Yersinia pestis*	48	94	Q7CH99.1	MFS transporter	
6878630	6880351	-	*DFER_RS28560*	*Dfer_5724*	WP_015815152.1	*Burkholderia cepacia* FAD glucose dehydrogenase (Chain A)	24	78	PMID: 36473944	Sugar dehydrogenase	
6880427	6880957	-	*DFER_RS28565*	*Dfer_5725*	WP_015815153.1	Predicted gluconate 2-dehydrogenase subunit 3	-	-	-	Sugar dehydrogenase	
6881534	6882583	+	*DFER_RS28570*	*Dfer_5726*	WP_015815154.1	Aldo-keto reductase from *Rhizobium leguminosarum*	40	93	PMID: 36042239	Reductase	
6882607	6883245	-	*DFER_RS28575*	*Dfer_5727*	WP_015815155.1	Response regulator from *Deinococcus radiodurans*	40	28	PMID: 34285211	Transcription regulator	
6883992	6885932	+	*DFER_RS28580*	*Dfer_5728*	WP_015815156.1	Predicted GH (unk)-CBM32-Ig-Ig-Ig	-	-	-	GH	
6886039	6886536	+	*DFER_RS28585*	*Dfer_5729*	WP_015815157.1	None - unknown (165 aa)	-	-	-	-	
6886608	6886802	+	*DFER_RS28590*	*Dfer_5730*	WP_015815158.1	None - unknown (64 aa)	-	-	-	-	

Table S5. Matrix of sequence identity of selected CE15 members. Representative CE15 members from fungi (fuchsia) and bacteria (blue) shown alongside the members from *D. fermentans* (green).

	*Cu*GE	*St*GE2	*Df*CE15A	*Df*CE15B	*Df*CE15C	*Ot*CE15A	*Su*CE15C
*Cu*GE	100.0	48.8	24.2	23.6	27.2	25.8	23.6
*St*GE2	48.8	100.0	26.3	25.7	27.1	24.6	24.1
*Df*CE15A	24.2	26.3	100.0	38.3	34.9	40.6	38.2
*Df*CE15B	23.6	25.7	38.3	100.0	35.6	37.6	41.9
*Df*CE15C	27.2	27.1	34.9	35.6	100.0	44.9	42.3
*Ot*CE15A	25.8	24.6	40.6	37.6	44.9	100.0	48.6
*Su*CE15C	23.6	24.1	38.2	41.9	42.3	48.6	100.0

Supplementary figures

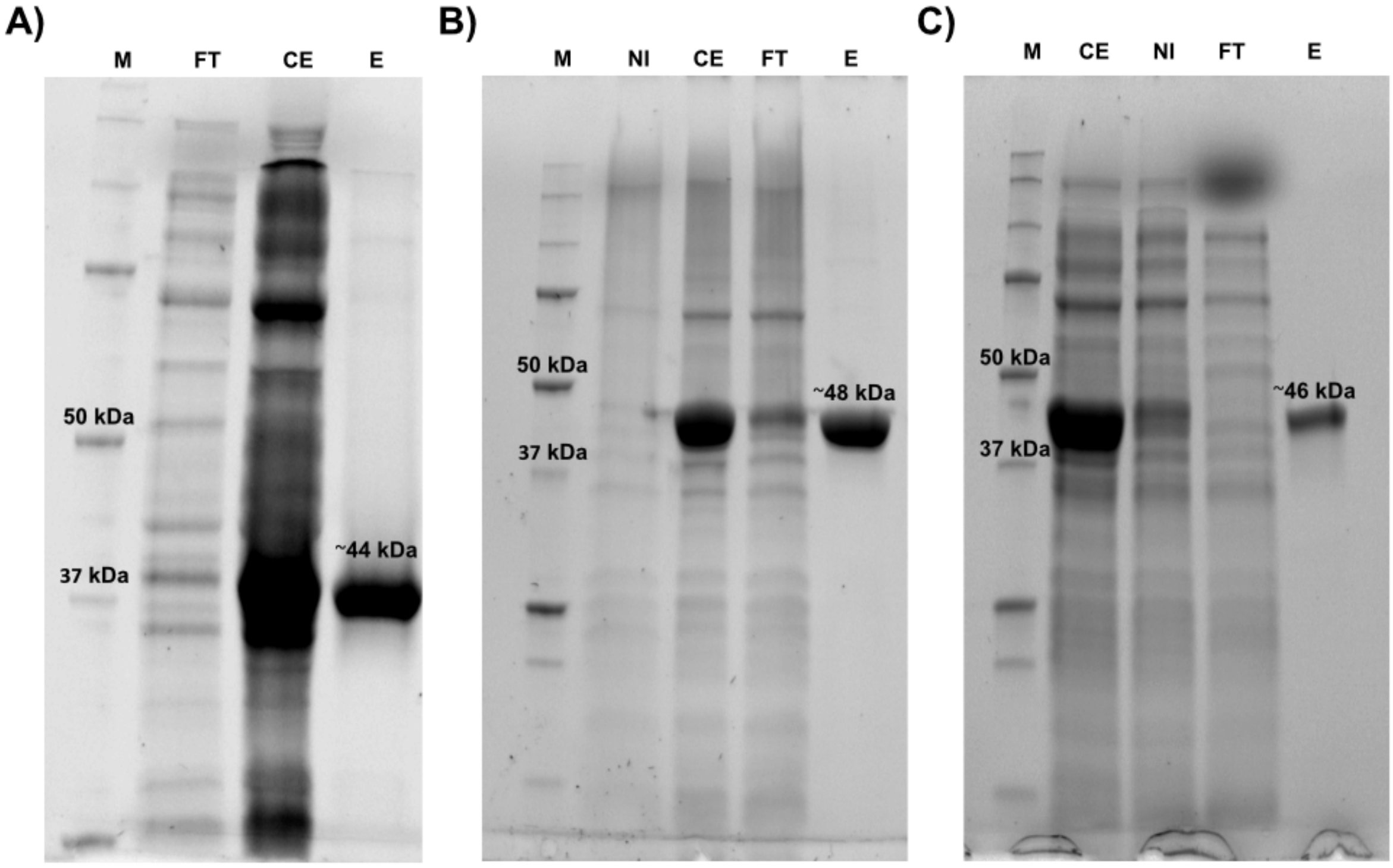

Fig. S1: SDS-PAGE of enzyme purification. SDS-PAGE gel images of the purification of A) *Df*CE15A, B) *Df*CE15B, and C) *Df*CE15C. Gel images contain a protein marker (M, Bio-rad Precision Plus Protein unstained standard), crude extract of induced cells (CE), crude extract of uninduced cells (NI), collected flow-through from the IMAC column loading (FT), and eluted fraction after the IMAC purification (E).

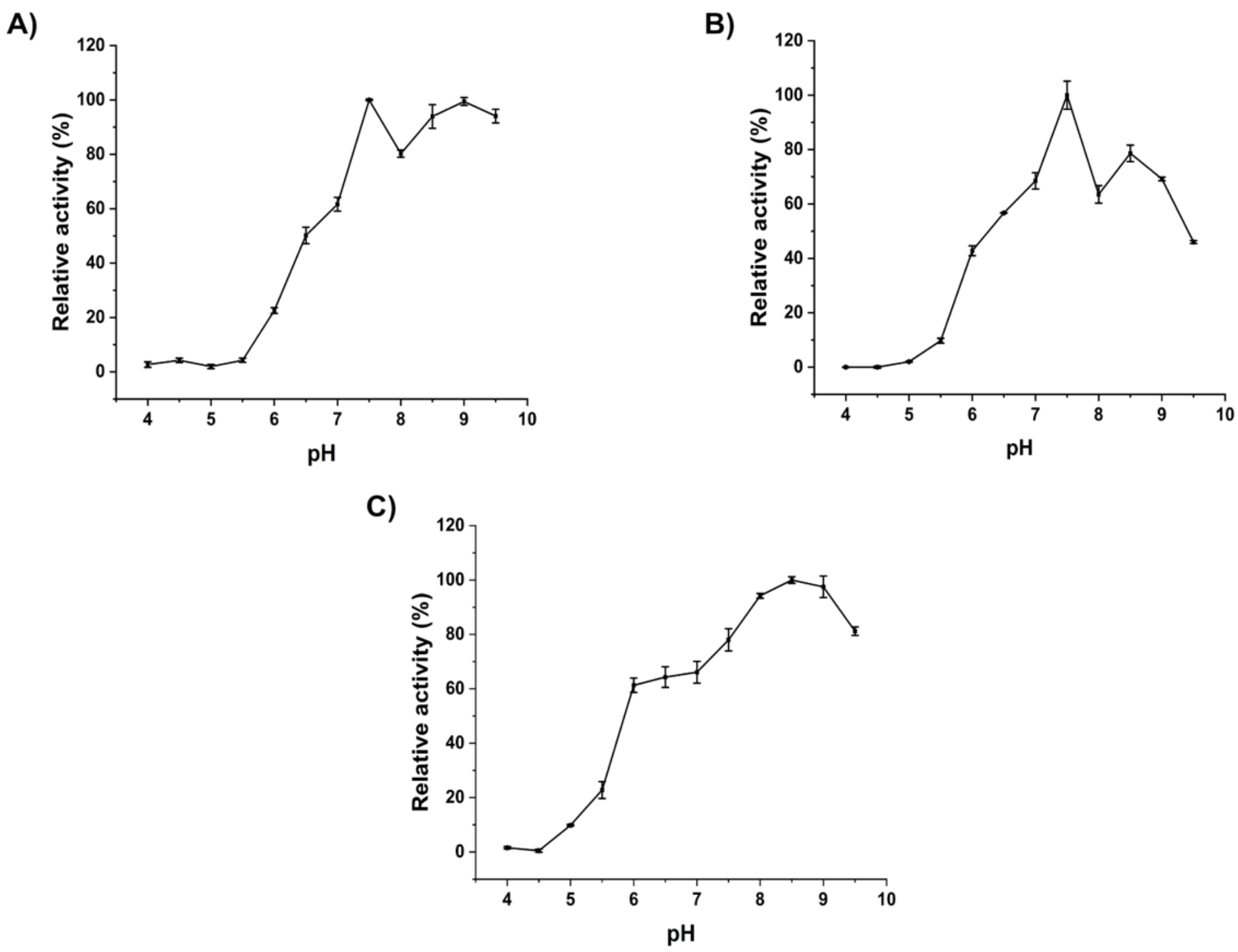

Fig. S2: Dependency of pH on BnzGlcA hydrolysis catalyzed by *Df*CE15A (A), *Df*CE15B (B) and *Df*CE15C (C). Specific activity with 2 mM BnzGlcA at different pH values was measured in three-component buffer as described in the methods. Error bars represent standard errors taken from duplicate measurements with the activity resulting in the highest activity for each enzyme taken as 100%. The rate of ester autohydrolysis increases with increasing pH values, and above pH 9.5 the high rate of autohydrolysis makes accurate determination of the enzymatic cleavage rate unreliable.

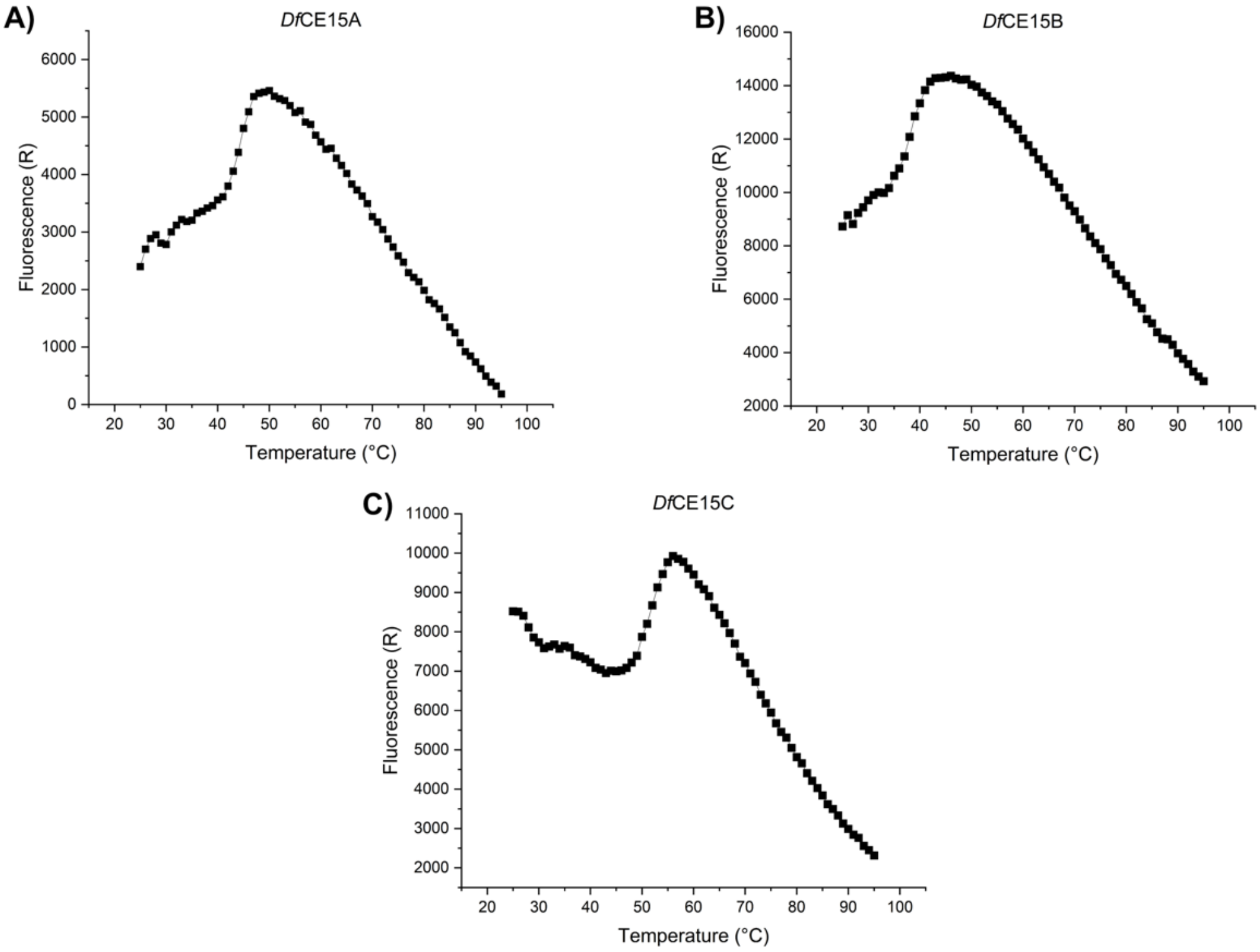

Fig. S3: Thermal shift plots of *Df*CE15s. Assay mixtures contained 5-10 µM of proteins with the temperature increasing by 1 °C/min and changes in fluorescence were quantified relative to a no protein control.

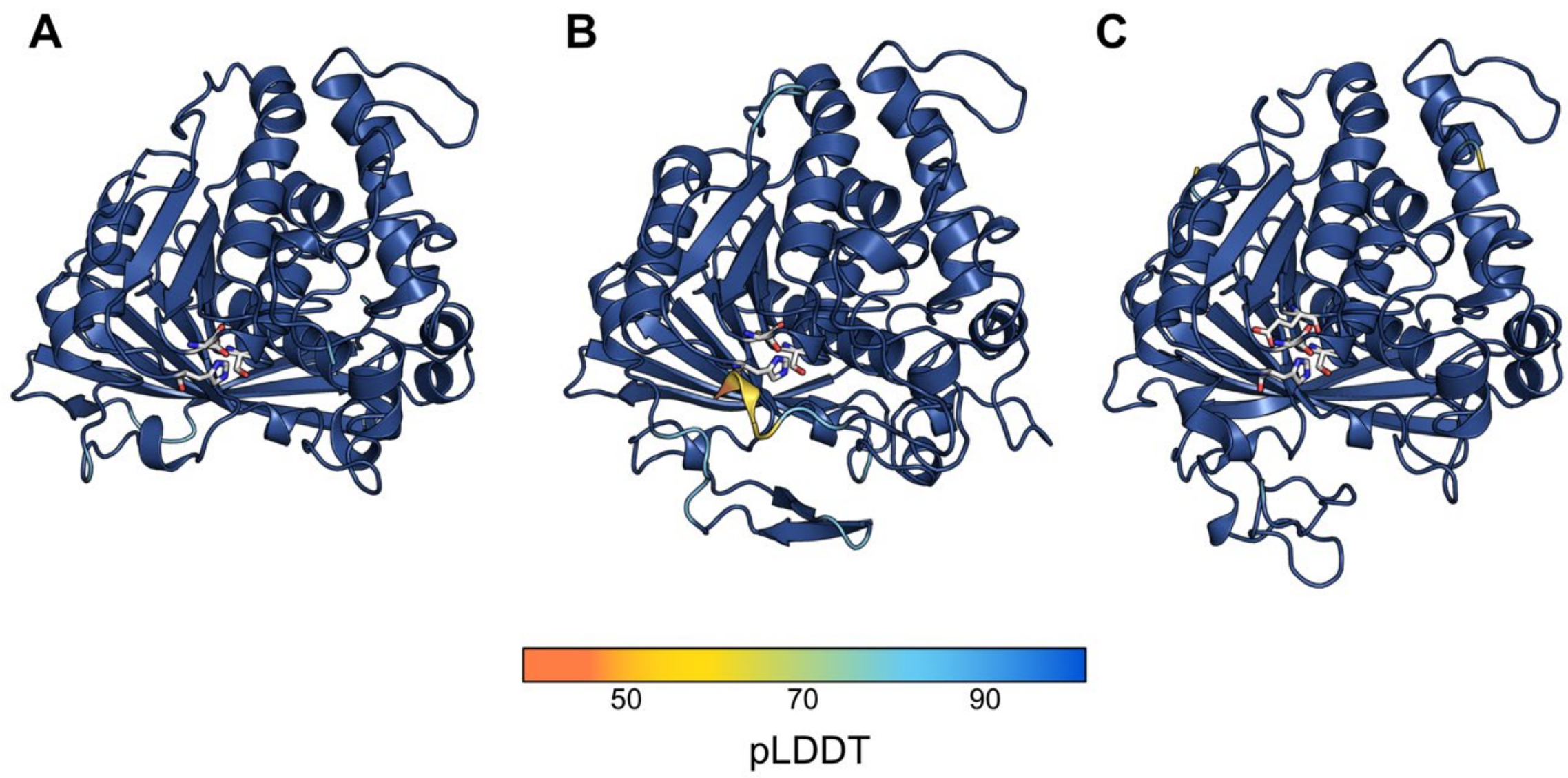

Fig. S4: The overall predicted structures of *Df*CE15A (A), *Df*CE15B (B) and *Df*CE15C (C), coloured relative to the pLDDT confidence values in the colour bar. The predictions are made using AlphaFold (Varadi et al. 2022). Note that all models have a very high confidence score.

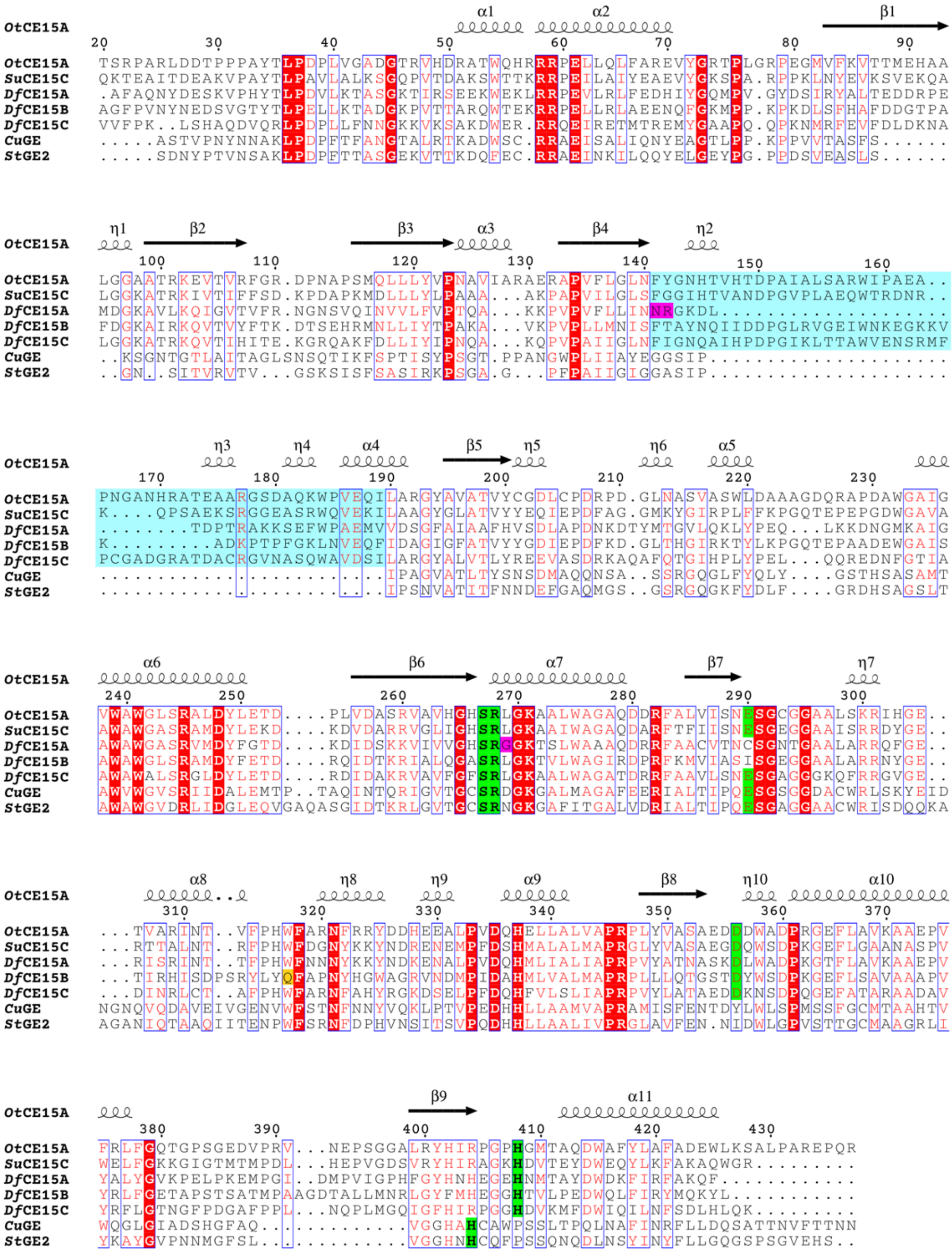

Fig. S5: Multiple sequence alignment of the *Df*CE15 enzymes and selected previously characterized CE15 members. The alignment contains sequences of characterized enzymes from both fungi (*C. unicolor*, *Cu*GE; and *S. thermophile* StGE2) and bacteria (*O. terrae*, *Ot*CE15A; and *S. usitatus*, *Su*CE15A). The residue numbering and secondary structural elements above the alignment are from *Ot*CE15A (PDB: 6GS0). Residues of the catalytic triad and oxyanion stabilizing arginine are shaded in green. Note that, while both bacterial and fungal catalytic histidine residues align structurally, they do not align by primary sequence alignment. Further, fungal CE15 enzyme catalytic acidic residues are found at the position equivalent to residue 290 in *Ot*CE15A, while in bacterial enzymes the acid is found at the position equivalent to residue 356 in *Ot*CE15A, and some bacteria contain both. "Region 2", found in bacterial members is highlighted in cyan. Residues targeted for substitution are highlighted in magenta for *Df*CE15A and orange for *Df*CE15B.

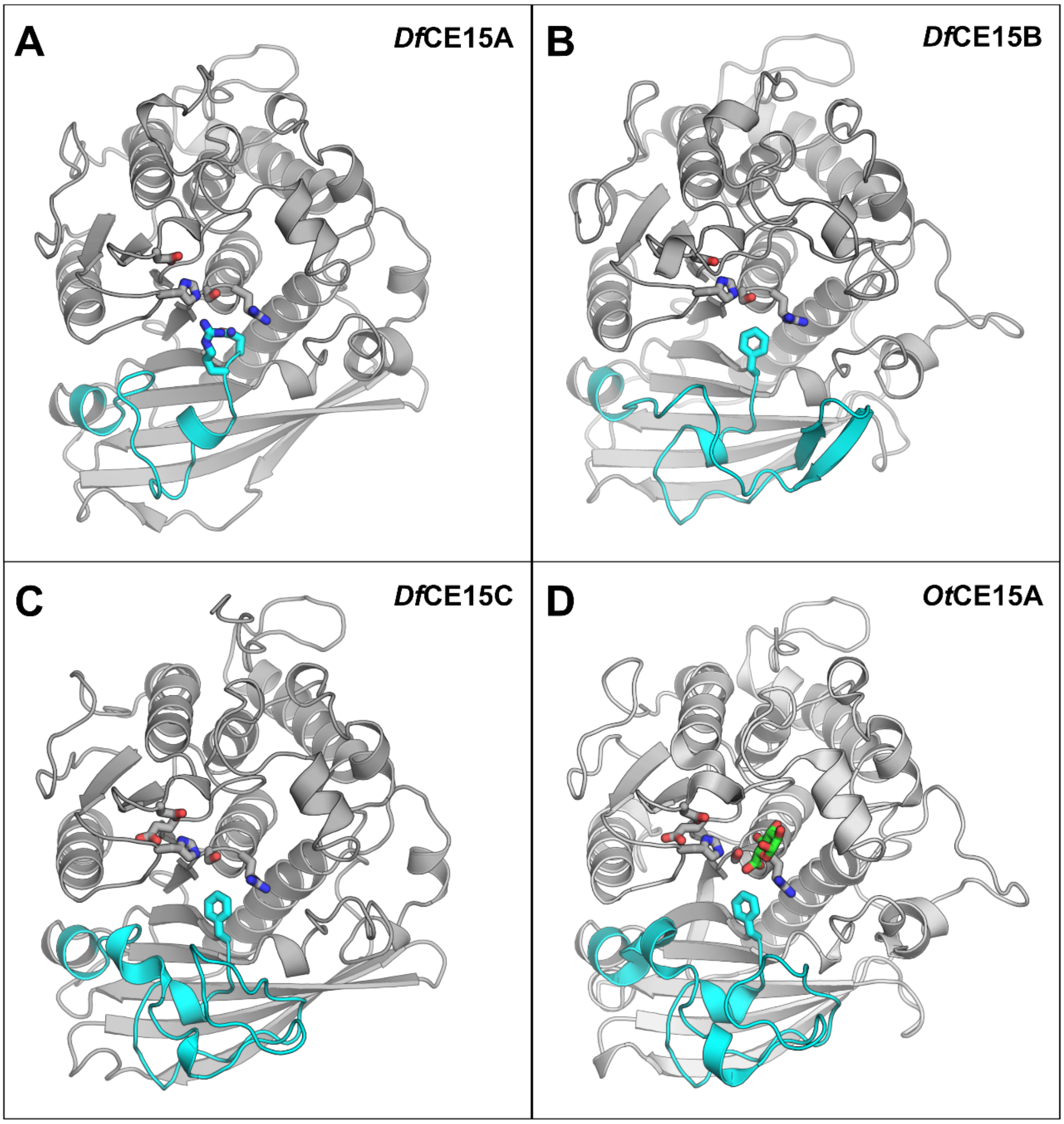

Fig. S6: Comparison of the overall folds of the *Df*CE15 protein models. The predicted models of *Df*CE15A (A), *Df*CE15B (B), *Df*CE15C (C) and the experimentally determined model of *Ot*CE15A with the bound glucuronate molecule shown as green sticks (D; PDB accession: 6SYR). The Region 2 in each structure is coloured in cyan. The figure was made using PyMOL 2.5.

References

Altschul SF, Gish W, Miller W, Myers EW, Lipman DJ (1990) Basic local alignment search tool. J Mol Biol 215(3):403-10, doi:10.1016/S0022-2836(05)80360-2

Consortium U (2023) UniProt: the Universal Protein Knowledgebase in 2023. Nucleic Acids Res 51(D1):D523-D531, doi:10.1093/nar/gkac1052

van Kempen M, Kim SS, Tumescheit C, Mirdita M, Lee J, Gilchrist CLM, Söding J, Steinegger M (2023) Fast and accurate protein structure search with Foldseek. Nat Biotechnol 42:243-246, doi:10.1038/s41587-023-01773-0

Varadi M, Anyango S, Deshpande M, Nair S, Natassia C, Yordanova G, Yuan D, Stroe O, Wood G, Laydon A, Žídek A, Green T, Tunyasuvunakool K, Petersen S, Jumper J, Clancy E, Green R, Vora A, Lutfi M, Figurnov M, Cowie A, Hobbs N, Kohli P, Kleywegt G, Birney E, Hassabis D, Velankar S (2022) AlphaFold Protein Structure Database: massively expanding the structural coverage of protein-sequence space with high-accuracy models. Nucleic Acids Res 50(D1):D439-D444, doi:10.1093/nar/gkab1061
